# Supplementary material for: Synthesis, DFT Studies, Molecular Docking and Biological Activity Evaluation of Thiazole-Sulfonamide Derivatives as Potent Alzheimer’s Inhibitors
Source: Molecules. 2023 Jan 5;28(2):559. doi: 10.3390/molecules28020559 (PMC9860845; doi:10.3390/molecules28020559)
Supplement: Supplementary file 1 [file molecules-28-00559-s001.zip › molecules-2009690-supplementary.pdf]

Article

Synthesis, DFT Studies, Molecular Docking and Biological Activity Evaluation of Thiazole-Sulfonamide Derivatives as Potent Alzheimer's Inhibitors

Shoaib Khan [1], Hayat Ullah [2,*], Muhammad Taha [3], Fazal Rahim [1,*], Maliha Sarfraz [4], Rashid Iqbal [5], Naveed Iqbal [6], Rafaqat Hussain [1], Syed Adnan Ali Shah [7,8], Khurshid Ayub [9], Marzough Aziz Albalawi [10],
Mahmoud A. Abdelaziz [11], Fatema Suliman Alatawi [12] and Khalid Mohammed Khan [13]

[1] Department of Chemistry, Hazara University, Mansehra 21120, Pakistan
[2] Department of Chemistry, University of Okara, Okara-56130, Pakistan
[3] Department of Clinical Pharmacy, Institute for Research and Medical Consultations (IRMC), Imam
Abdulrahman Bin Faisal University, P.O. Box 1982, Dammam 31441, Saudi Arabia
[4] Department of Zoology, Wildlife and Fisheries, University of Agriculture Faisalabad, Sub-Campus,
Toba Tek Singh 36050, Pakistan
[5] Department of Agronomy, Faculty of Agriculture and Environment, The Islamia University of Bahawalpur, Bahawalpur-63100, *Pakistan*
[6] Department of Chemistry, University of Poonch, Rawalakot 12350, Pakistan
[7] Faculty of Pharmacy, Universiti Teknologi MARA Cawangan Selangor Kampus Puncak Alam,
Puncak Alam 42300, Selangor, Malaysia
[8] Atta-ur-Rahman Institute for Natural Product Discovery (AuRIns), Universiti Teknologi MARA Cawangan Selangor Kampus Puncak Alam, Puncak Alam 42300, Selangor, Malaysia
[9] Department of Chemistry, COMSATS University, Abbottabad Campus, Abbottabad 22060, KPK, Pakistan
[10] Department of Chemistry, Alwajh College, University of Tabuk, Tabuk 71491, Saudi Arabia
[11] Department of Chemistry, Faculty of Science, University of Tabuk, Tabuk 71491, Saudi Arabia; m_mahmoud@ut.edu.sa
[12] Department of Biochemistry, Faculty of Science, University of Tabuk, Tabuk 71491, Saudi Arabia
[13] H.E.J. Research Institute of Chemistry, International Center for Chemical and Biological Sciences, University of Karachi, Karachi 75270, Pakistan
* Correspondence: ayaanwazir366@gmail.com (H.U.); fazalstar@gmail.com (F.R.)

....

Supporting Information

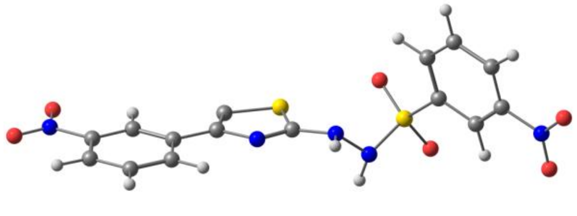

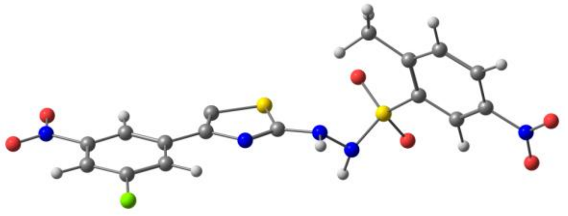

3	4

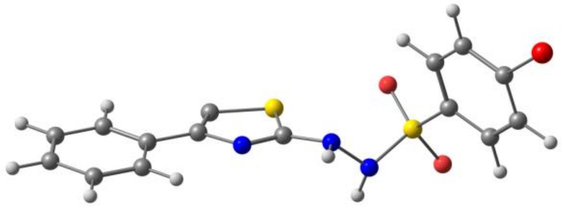

5

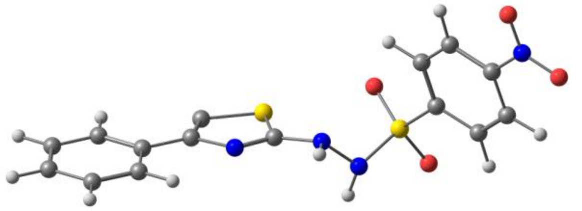

6

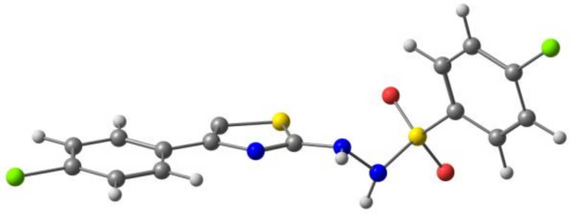

7

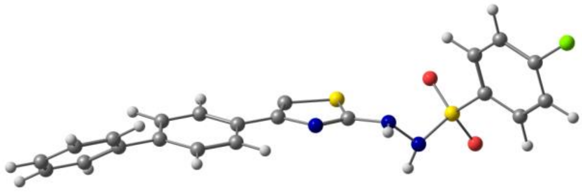

8

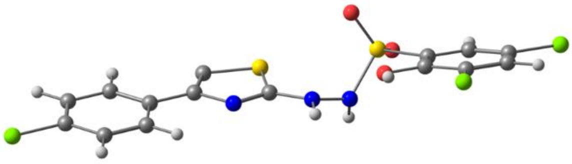

9

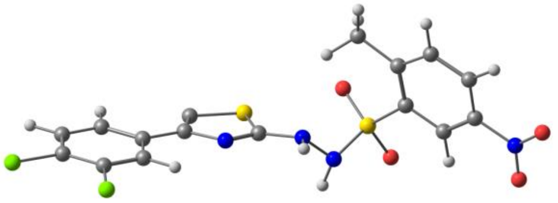

10

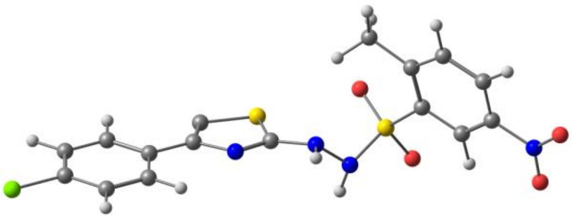

11

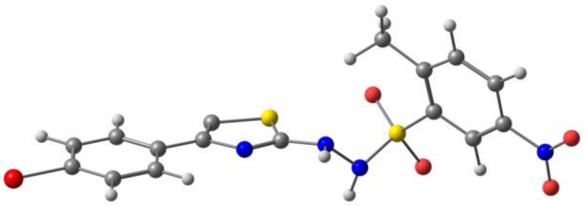

12

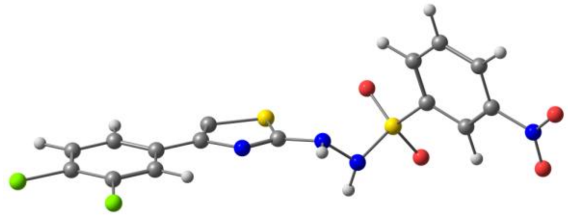

13

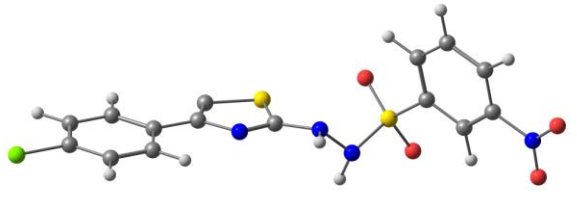

14

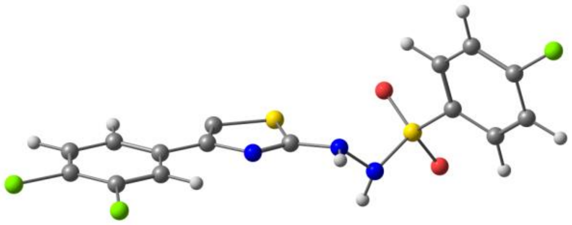

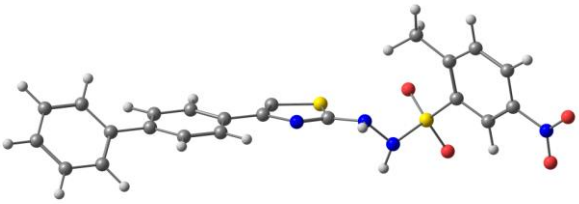

15	16
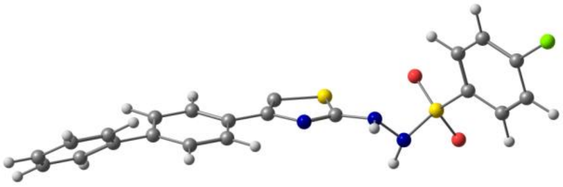	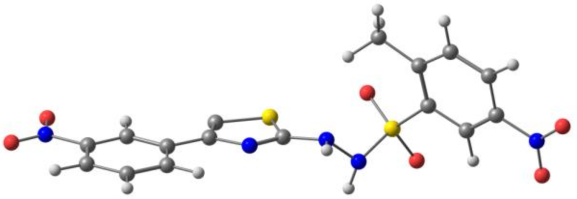
17	18
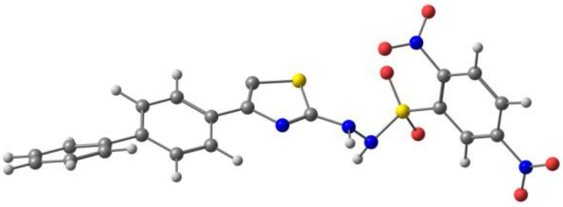	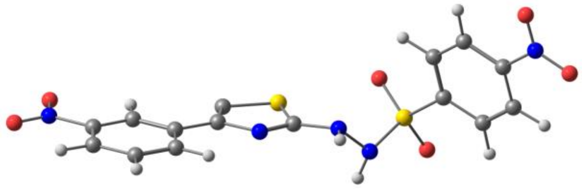
19	20

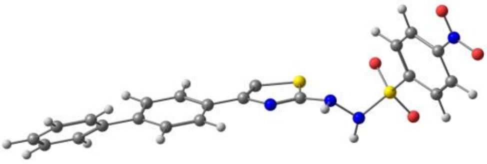

21

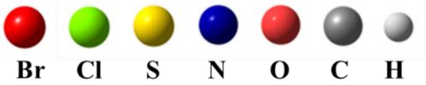

Figure-S1: Optimized structures of representative thiazole bearing sulfonamide analogues (**3-21**) at the ωB97XD/6-31g(d,p) level of theory

-0.058	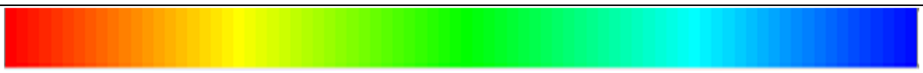	+0.058

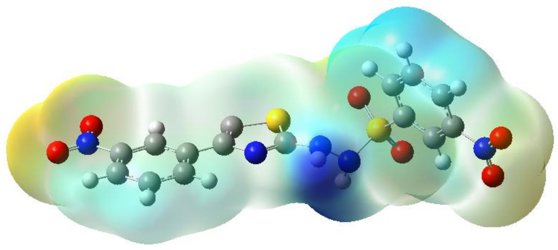	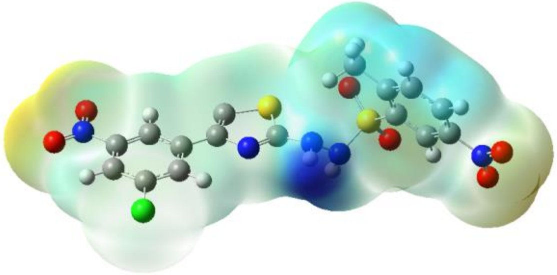
3	4

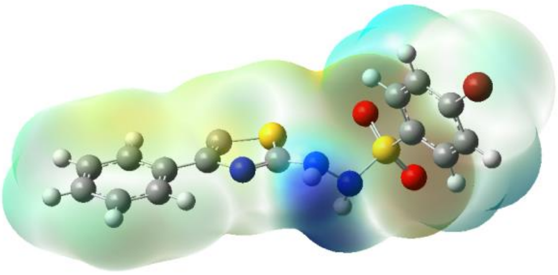

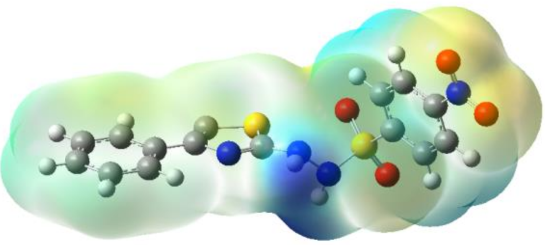

**5**	**6**

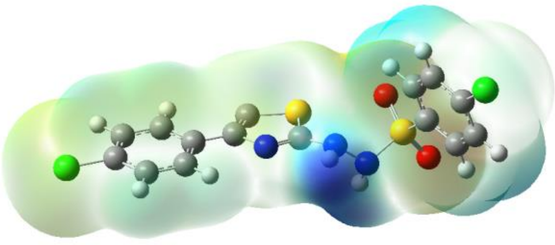

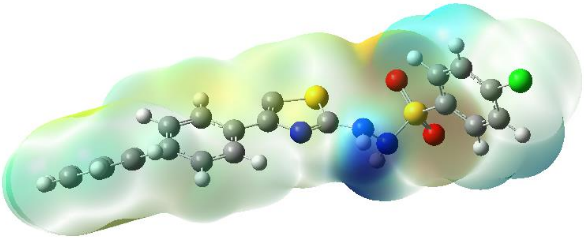

**7**	**8**

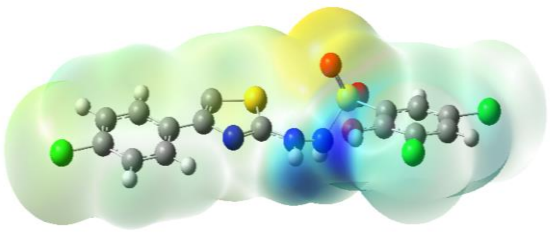

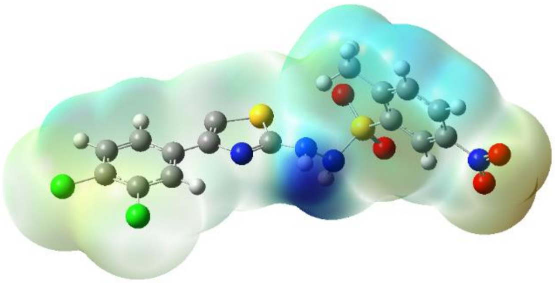

**9**	**10**

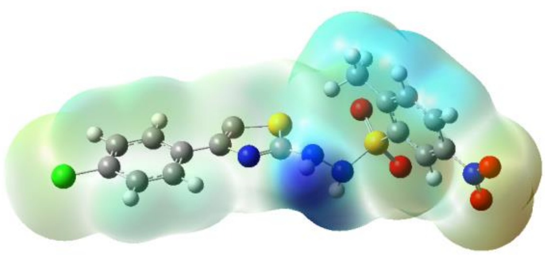

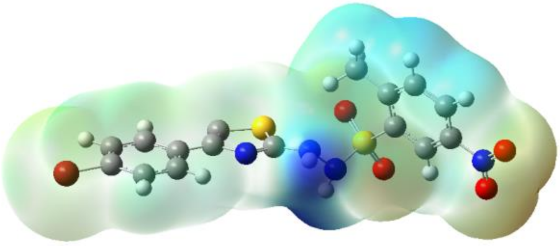

**11**	**12**

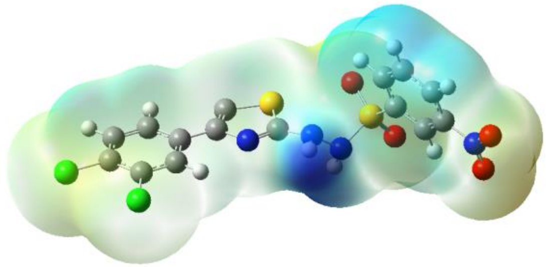

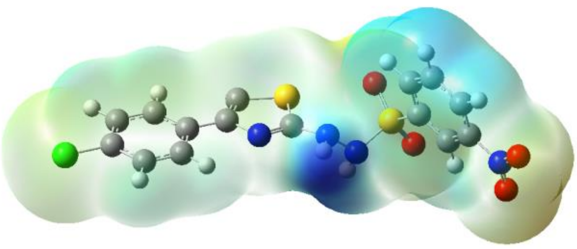

**13**	**14**

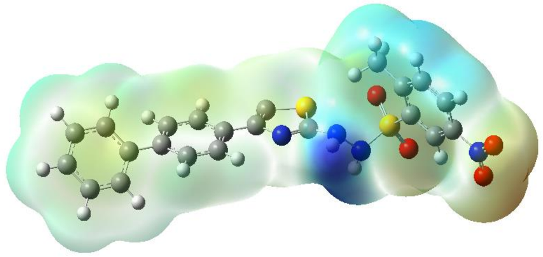	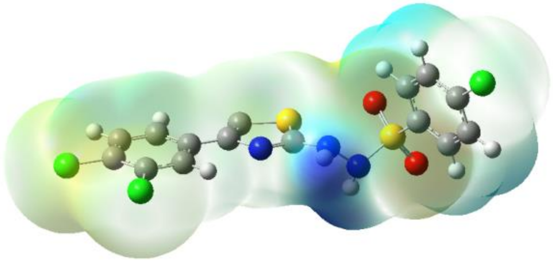
**15**	**16**
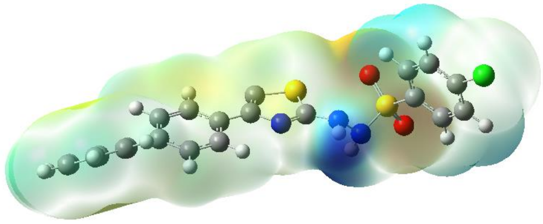	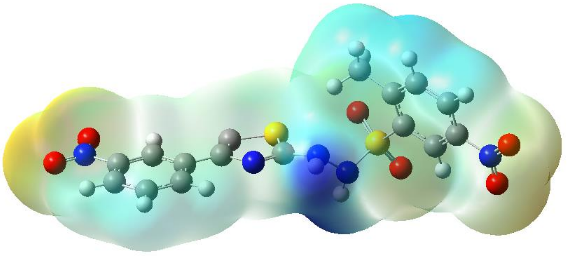
**17**	**18**
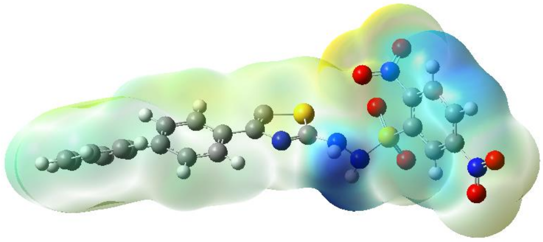	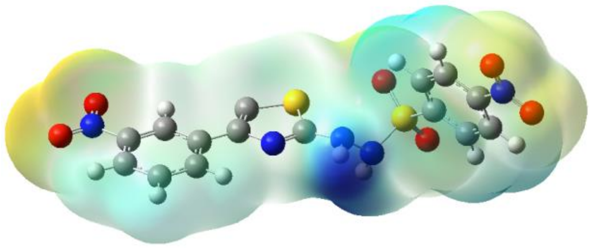
**19**	**20**

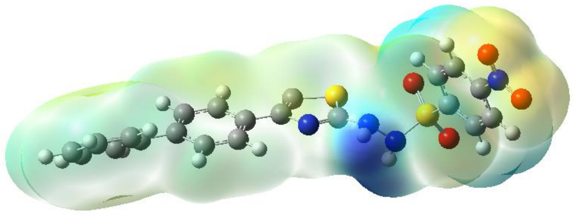

21

Figure-S2: Molecular Electrostatics Potential (MESP) of representative thiazole bearing sulfonamide analogues (**3-21**).

**HOMO**	**LUMO**

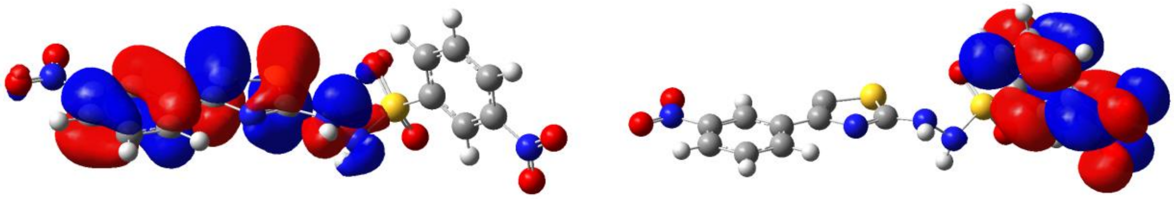

3

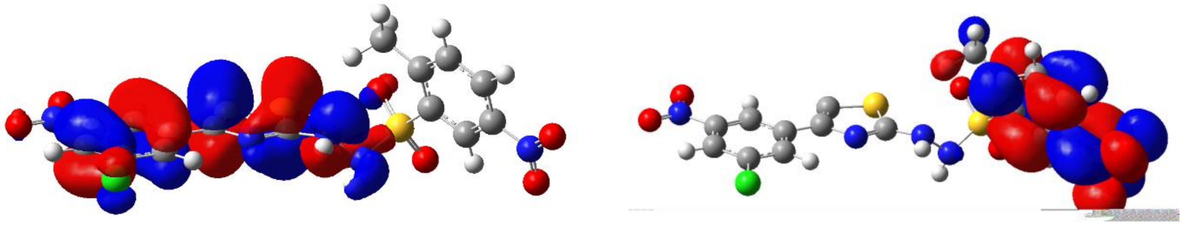

4

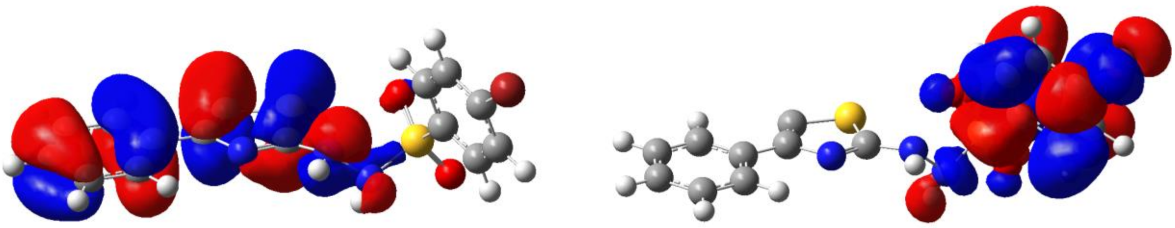

5

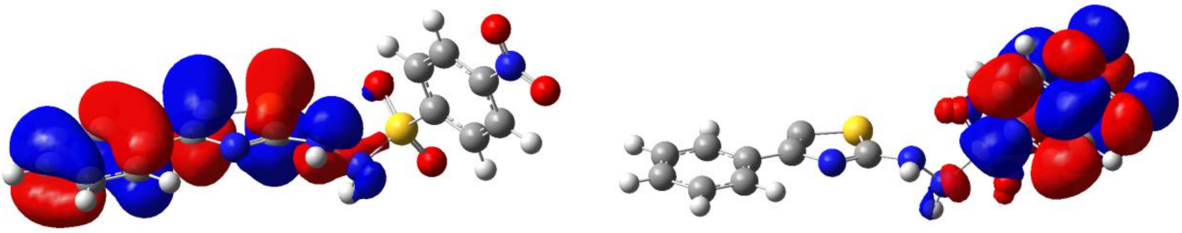

6

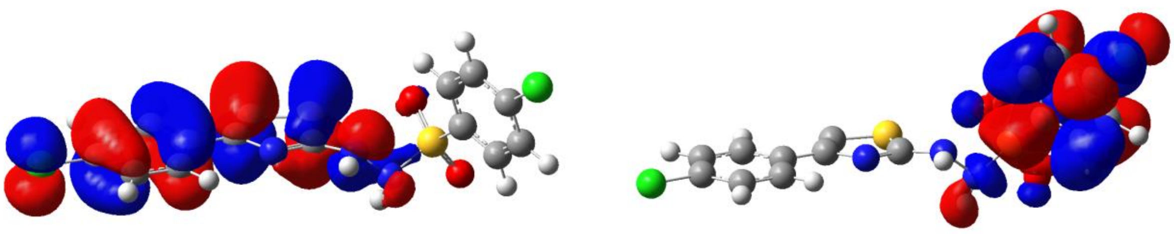

7

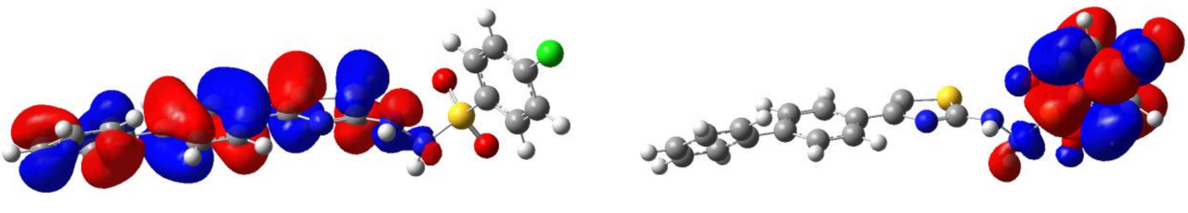

8

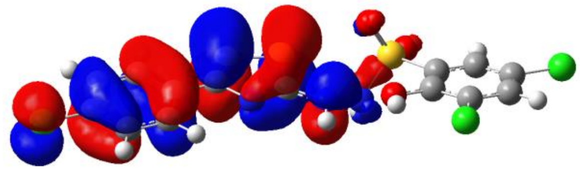 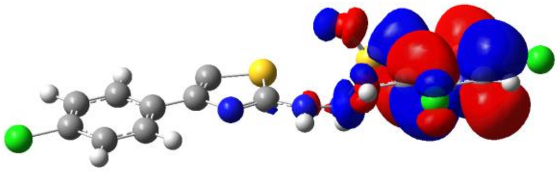

9

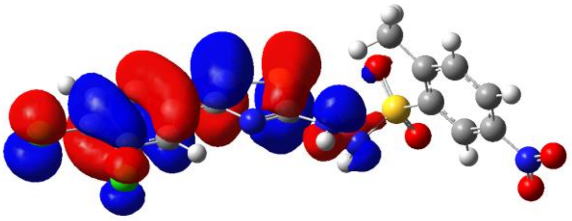 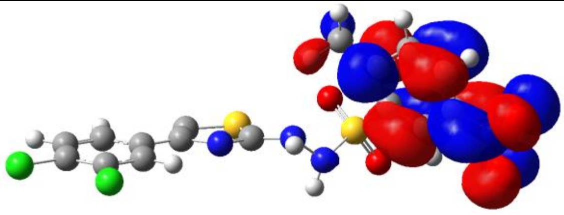

10

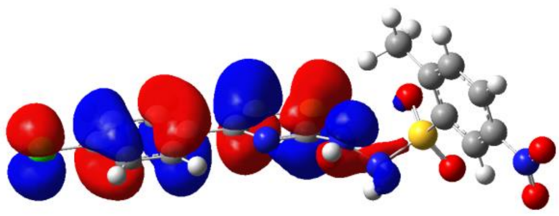 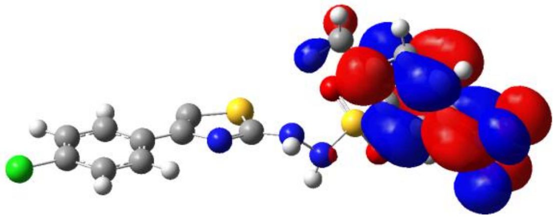

11

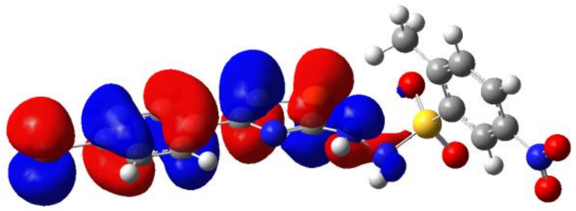 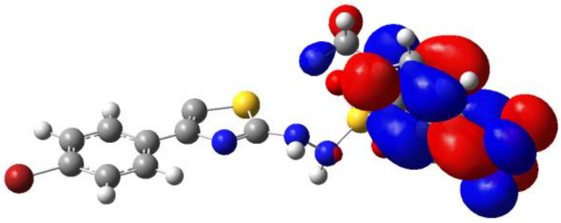

12

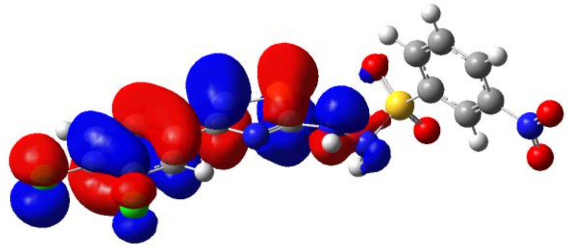 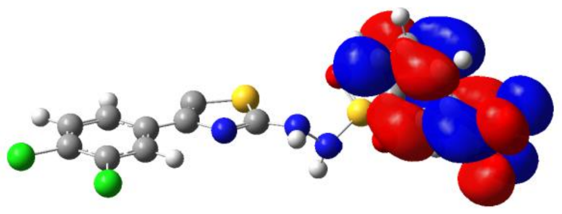

13

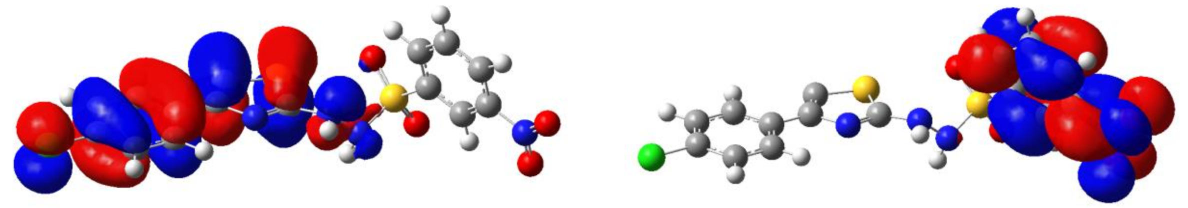

14

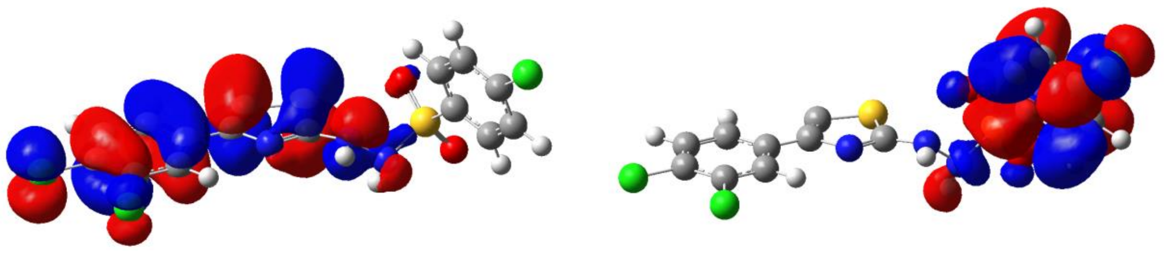

15

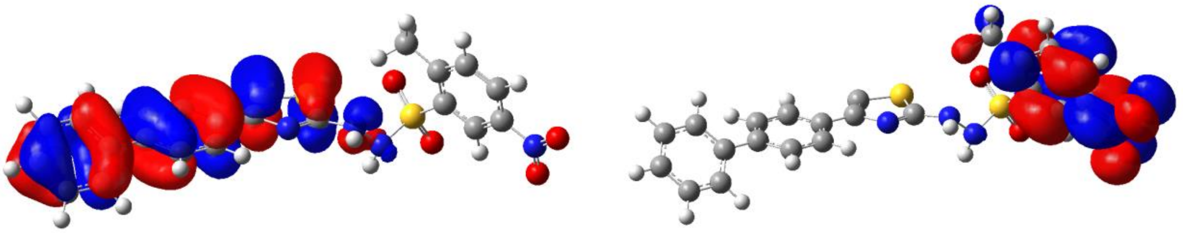

16

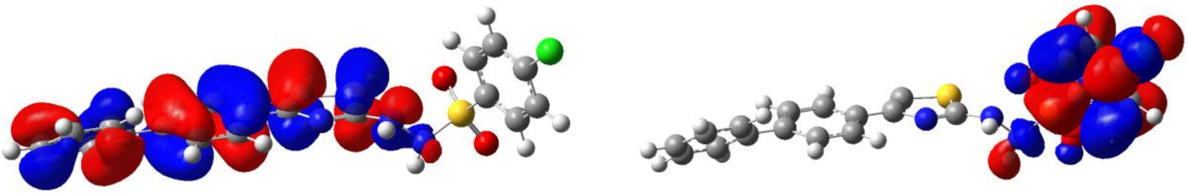

17

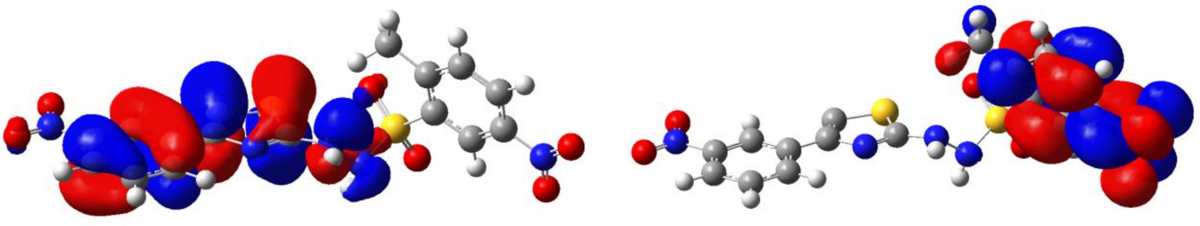

18

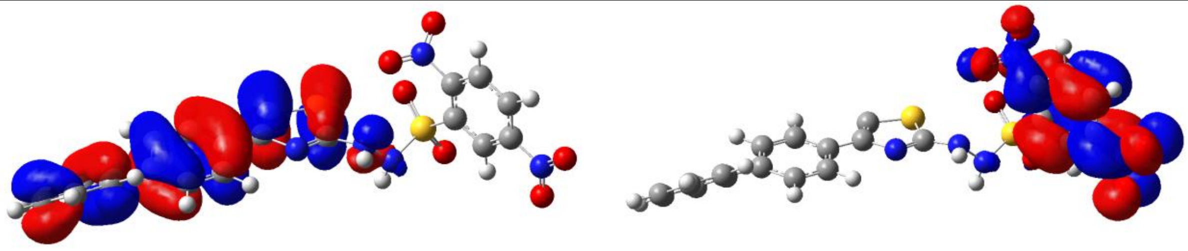

19	
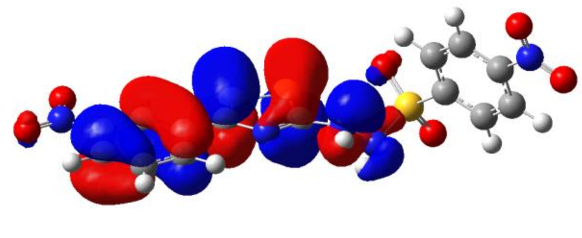	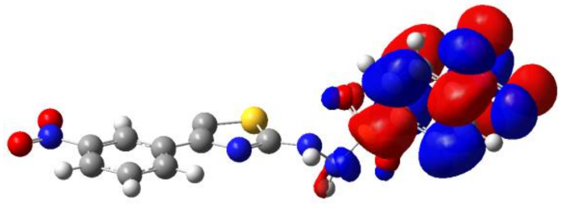
20	
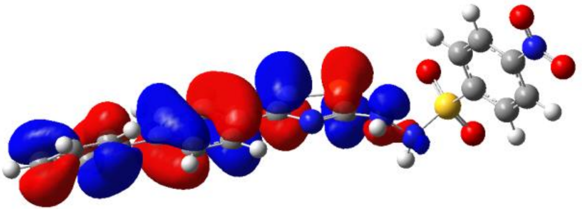	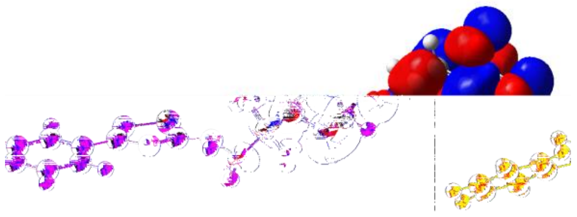
21	

Figure-S3: HOMO-LUMO orbital densities of studies thiazole bearing sulfonamide analogues (**3-21**).

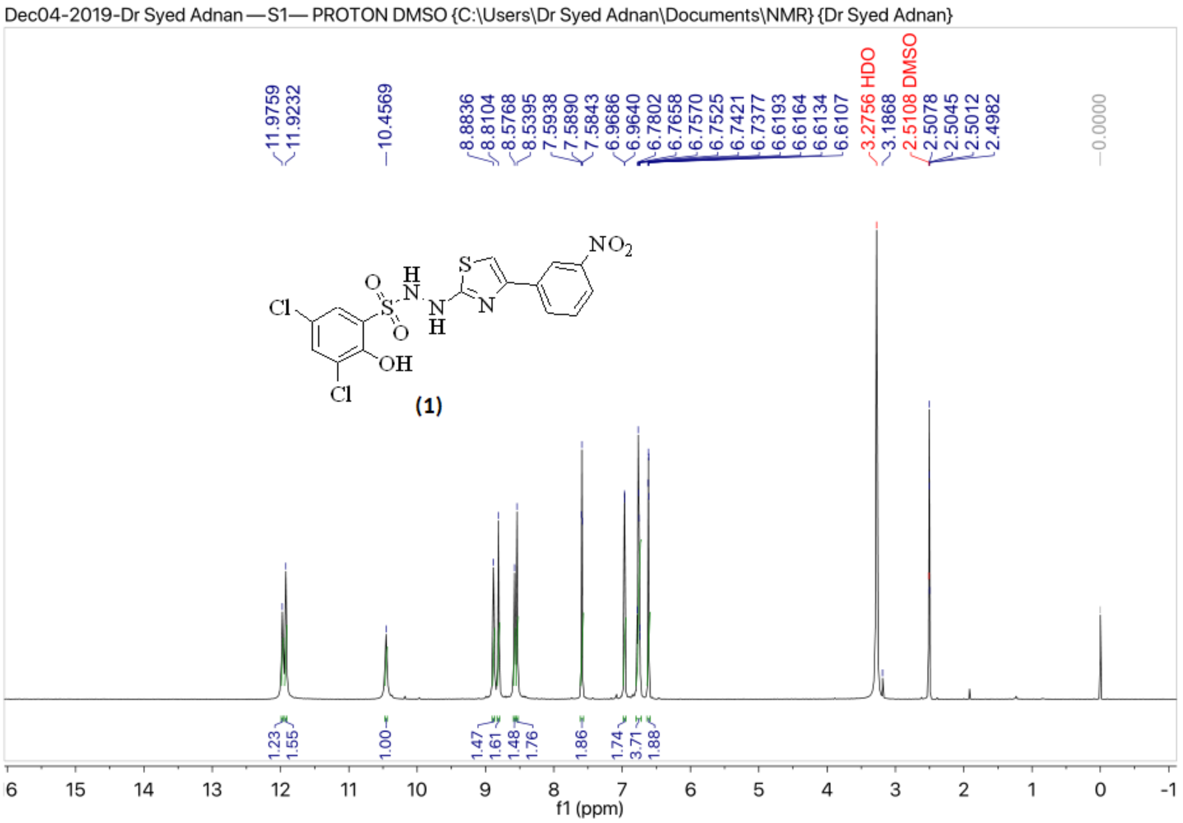

Figure-S4: Represent HNMR of analog 1

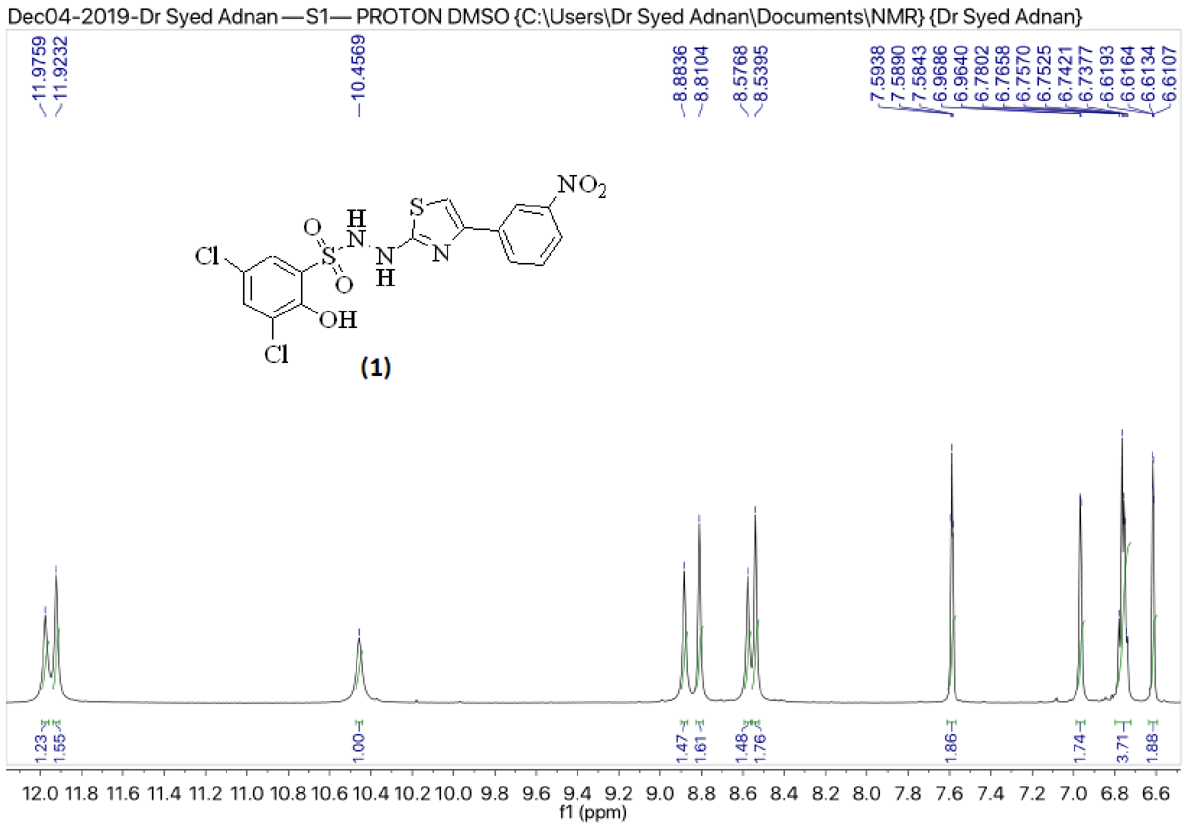

Figure-S5: Represent HR-HNMR of analog 1

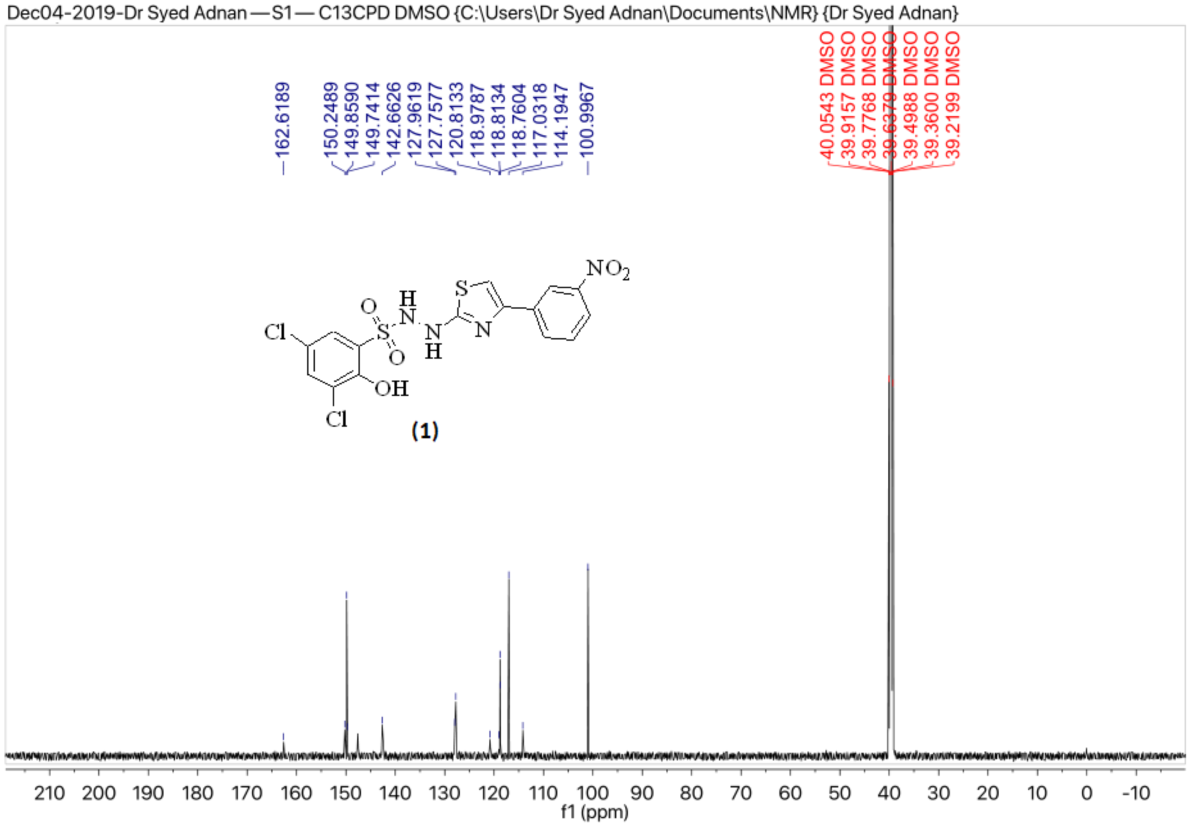

Figure-S6: Represent CNMR of analog 1

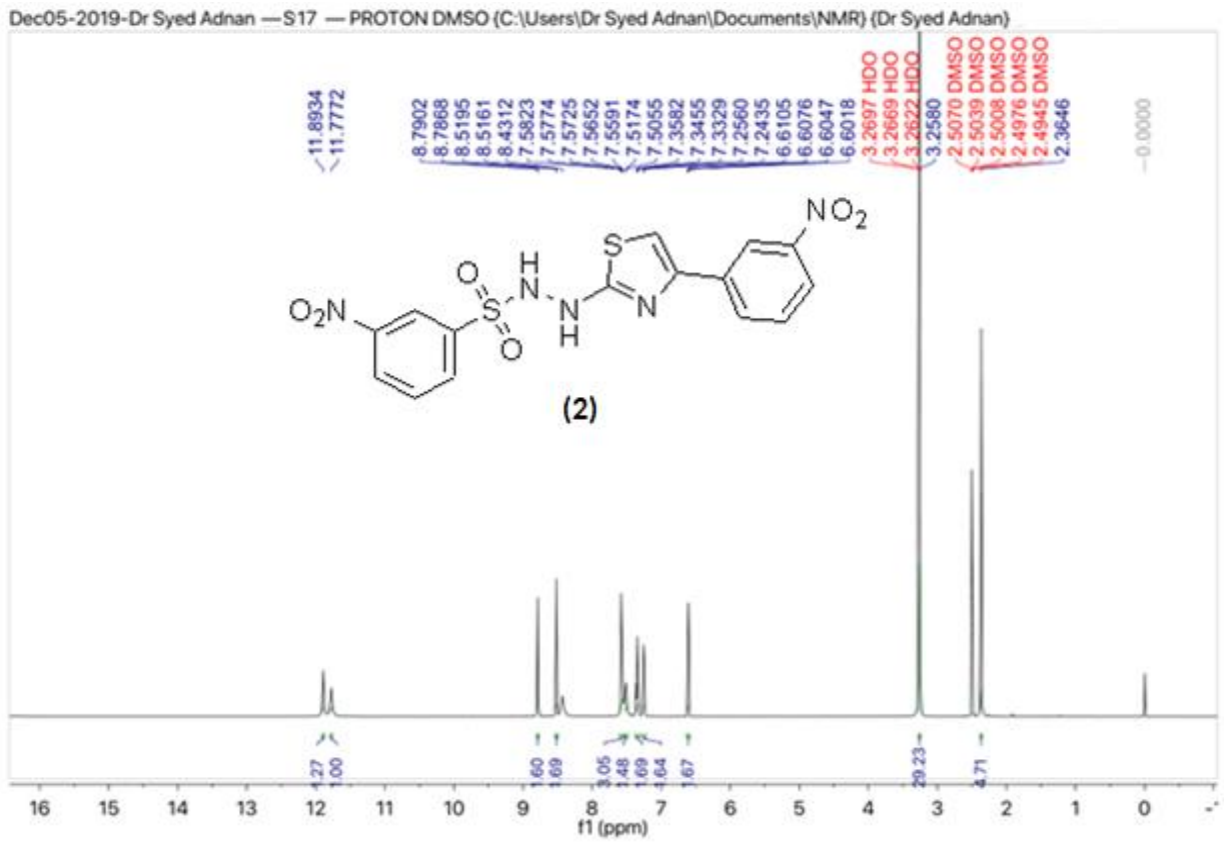

Figure-S7: Represent HNMR of analog 2

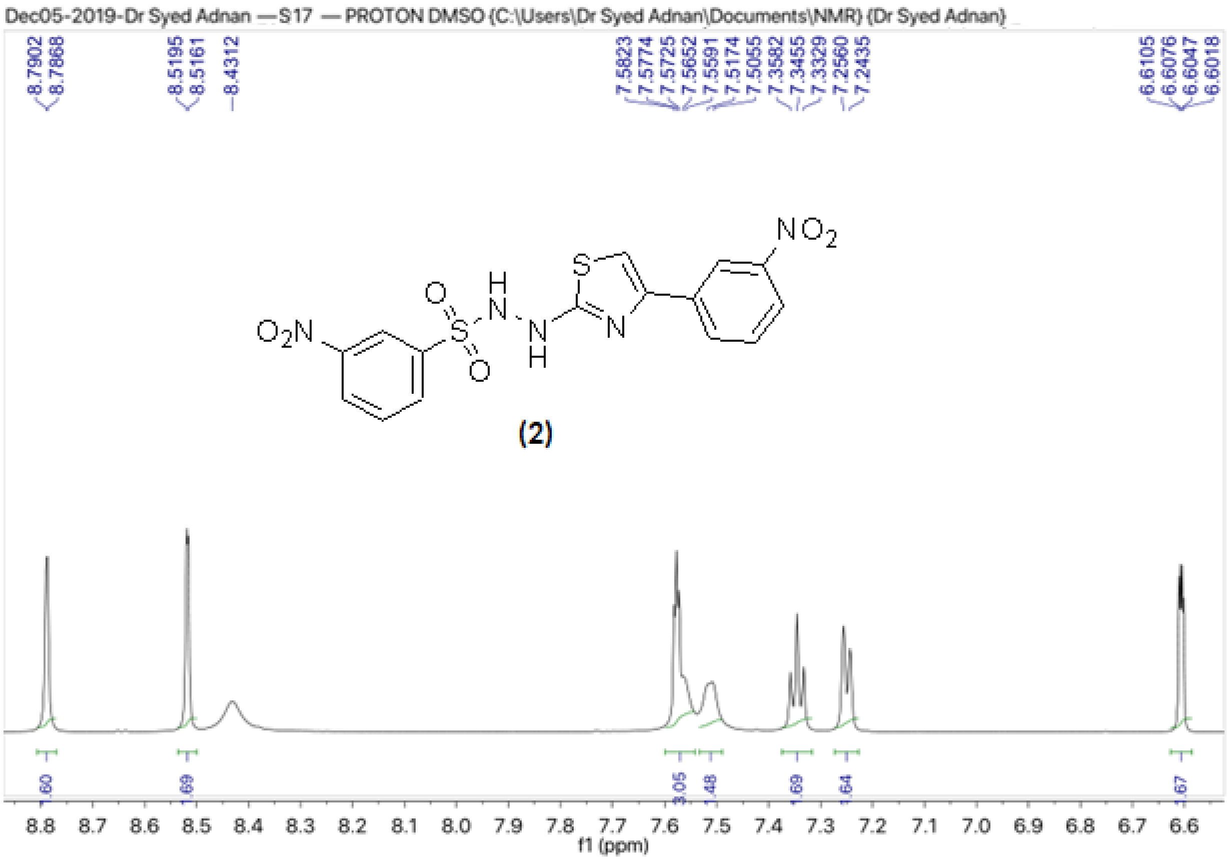

Figure-S8: Represent HR-HNMR of analog 2

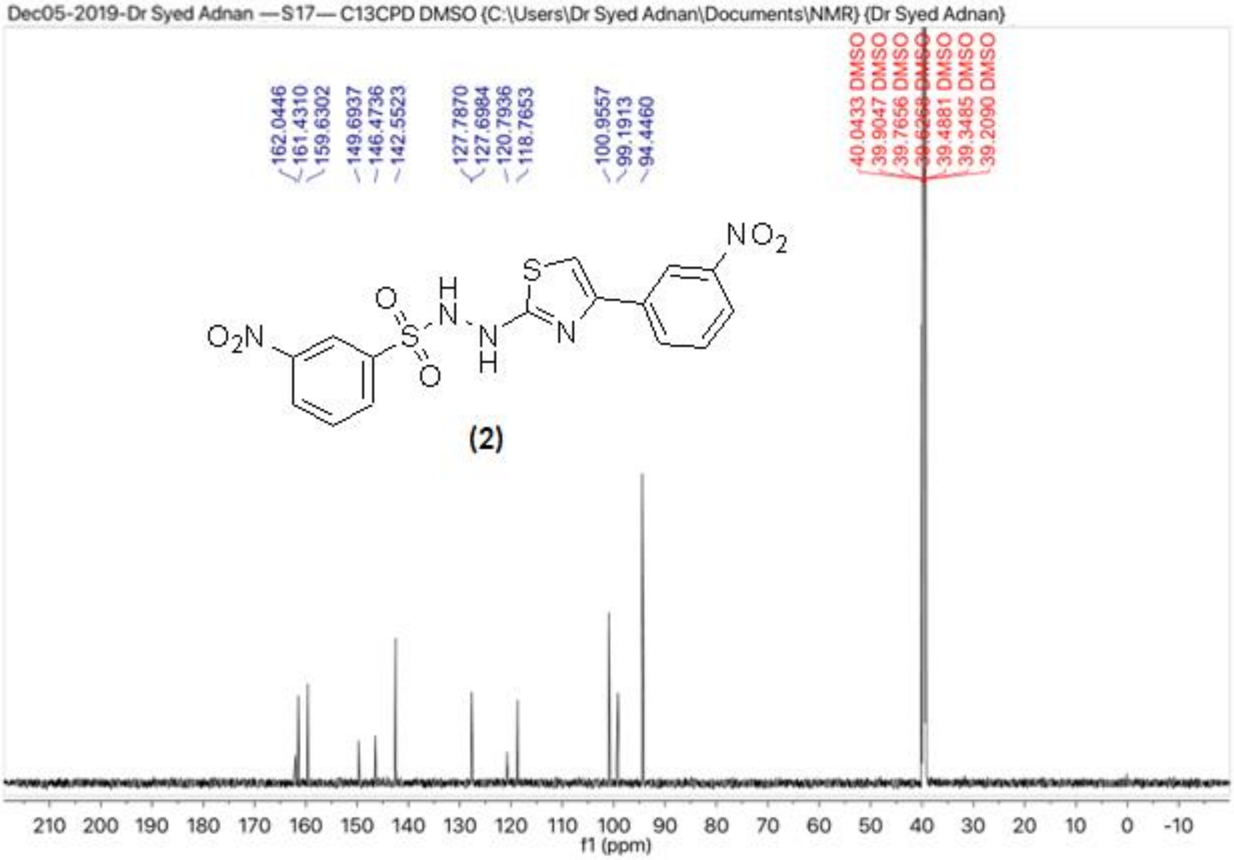

Figure-S9: Represent CNMR of analog 2

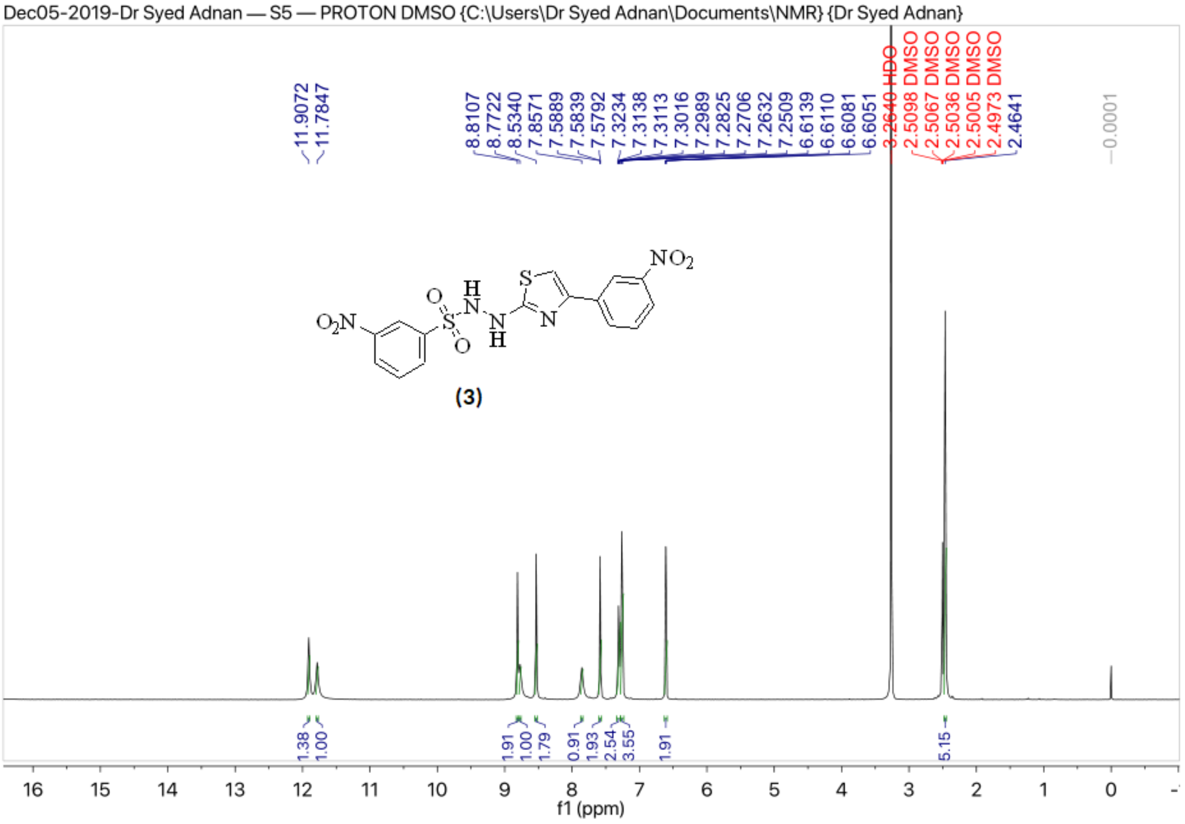

Figure-S10: Represent HNMR of analog 3

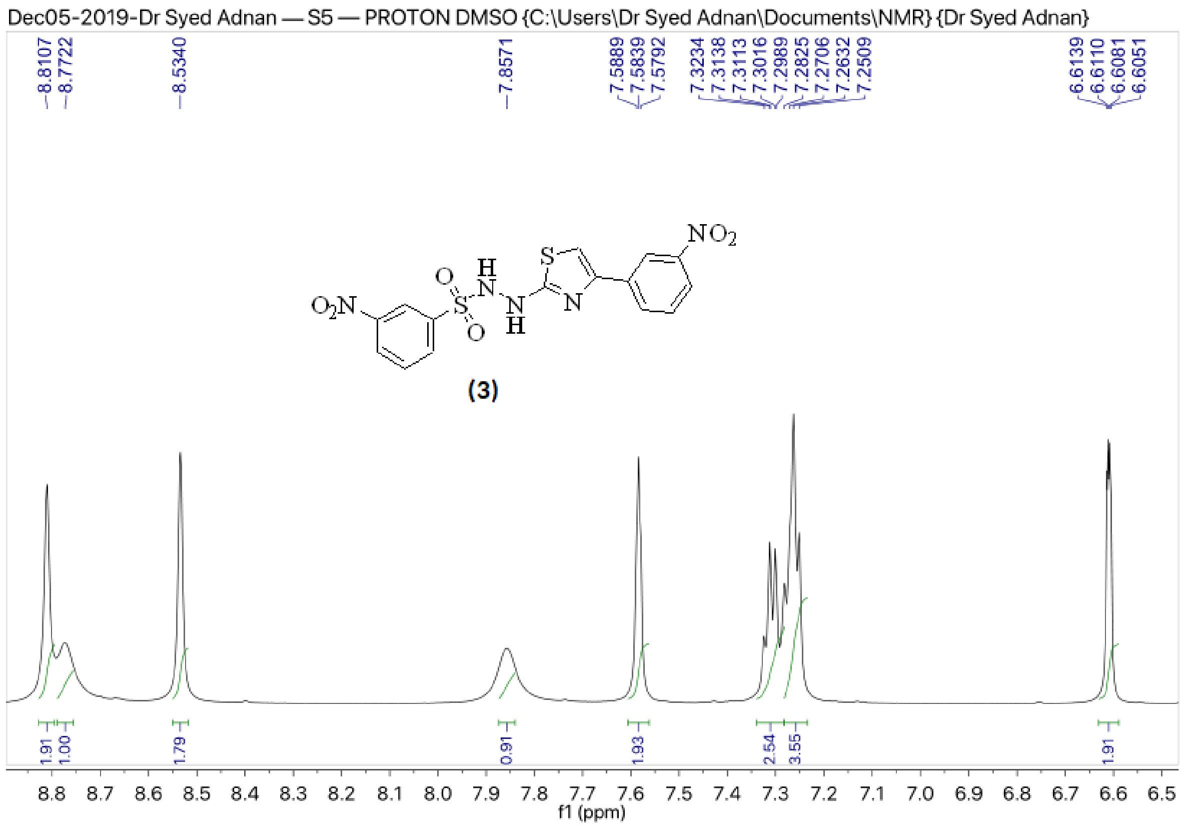

Figure-S11: Represent HR-HNMR of analog 3

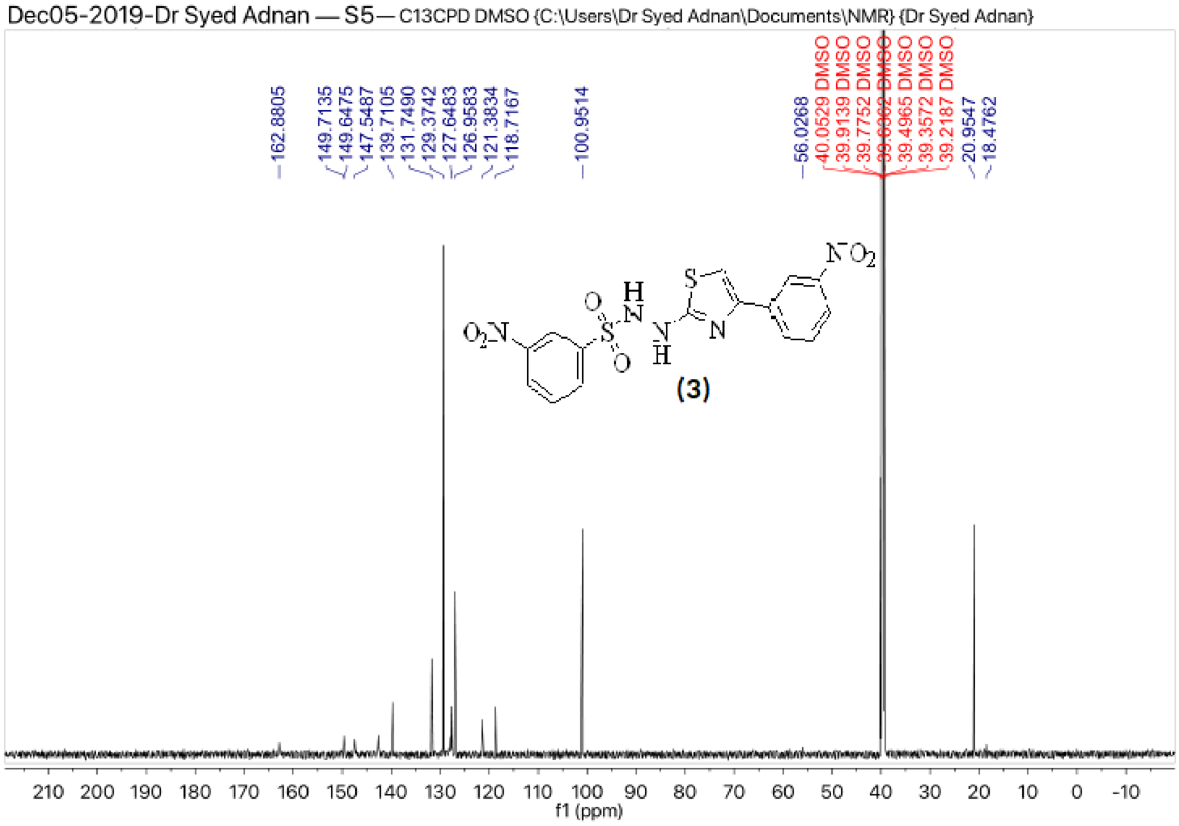

Figure-S12: Represent CNMR of analog 3

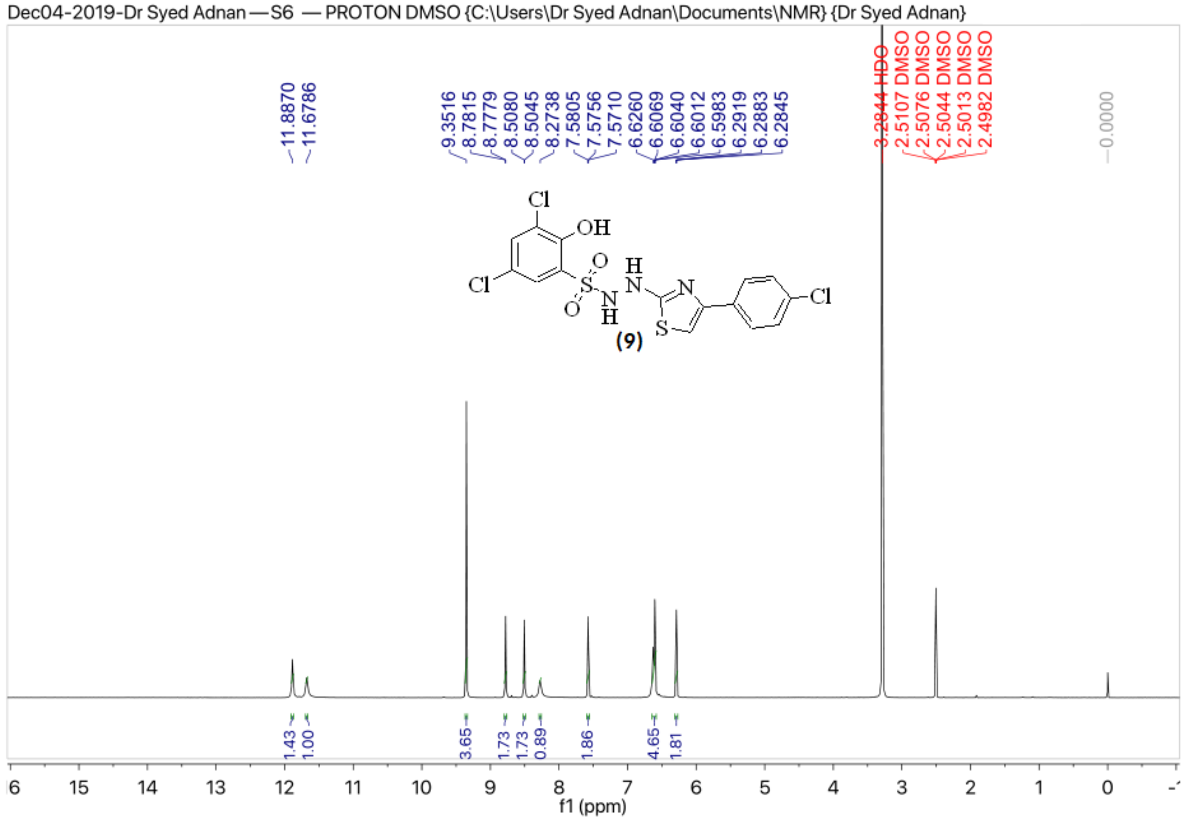

Figure-S13: Represent HNMR of analog 9

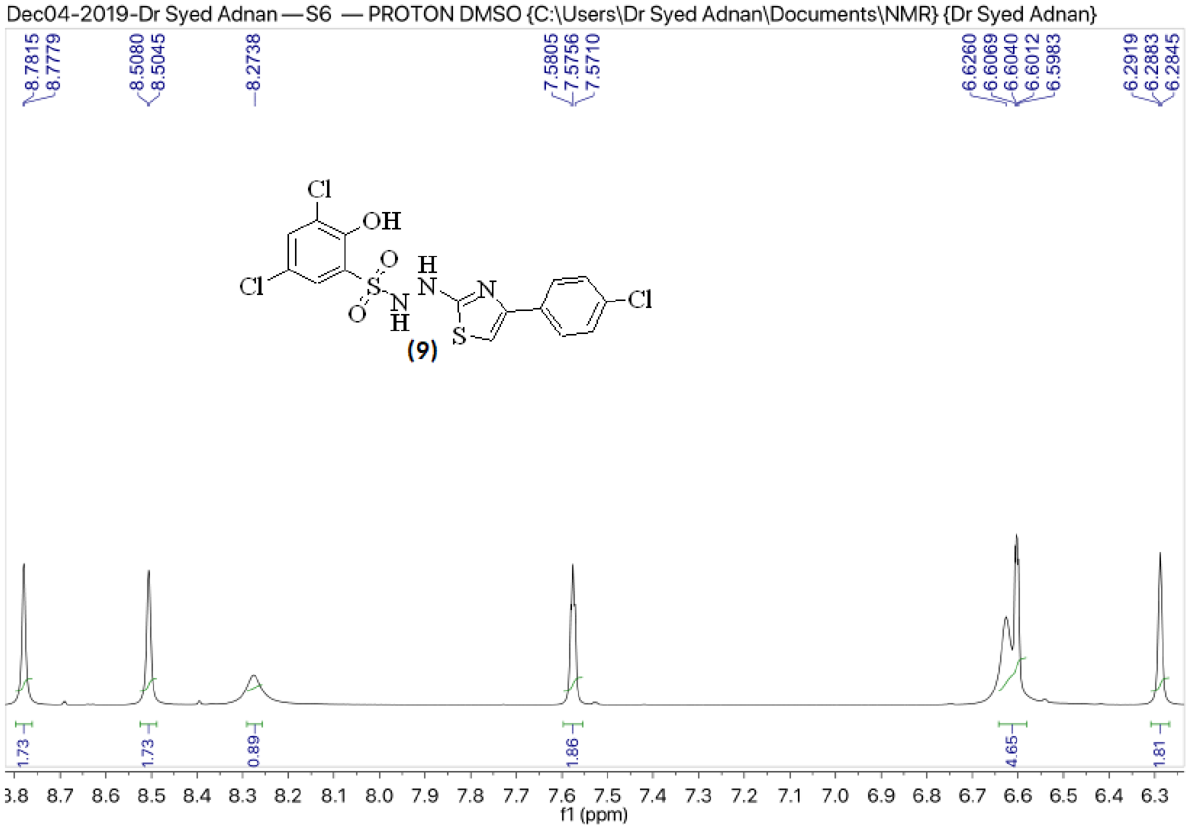

Figure-S14: Represent HR-HNMR of analog 9

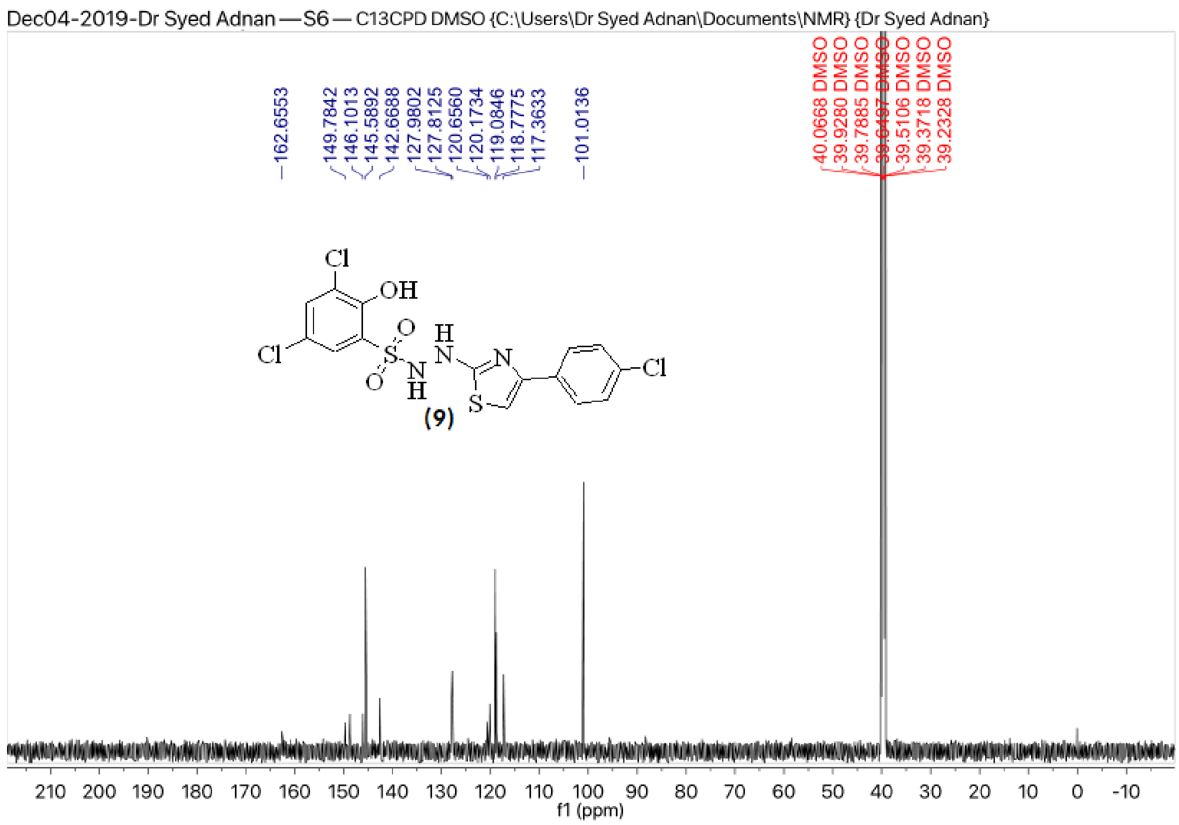

Figure-S15: Represent CNMR of analog 9

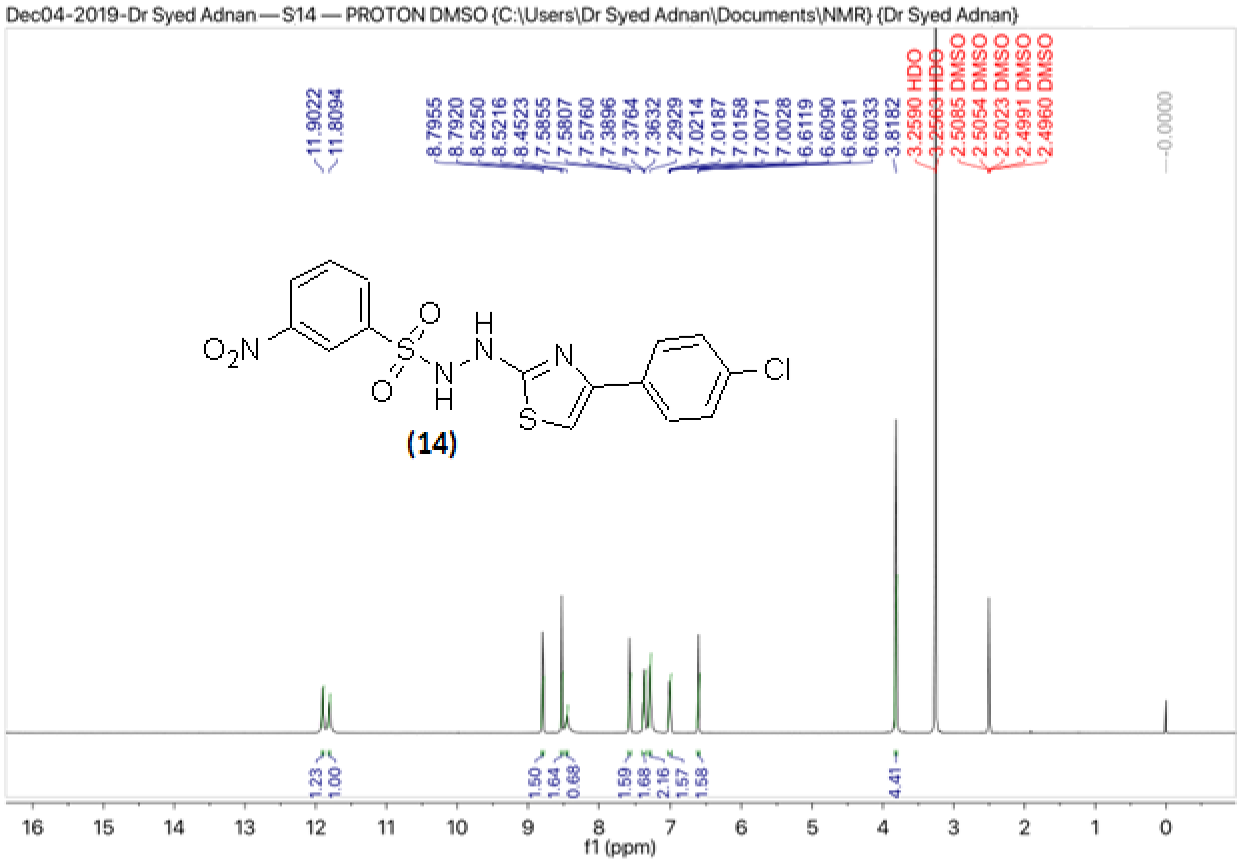

Figure-S16: Represent HNMR of analog 14

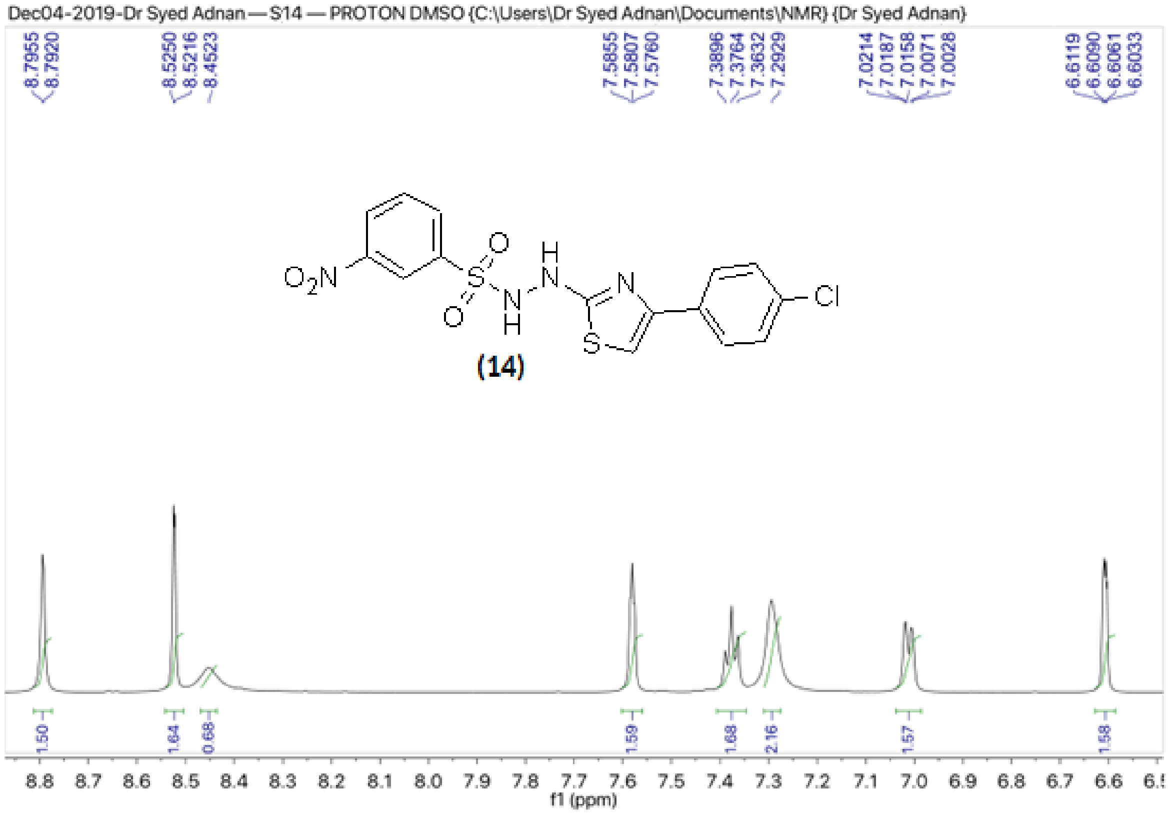

Figuire-S17: Represent HR-HNMR of analog 14

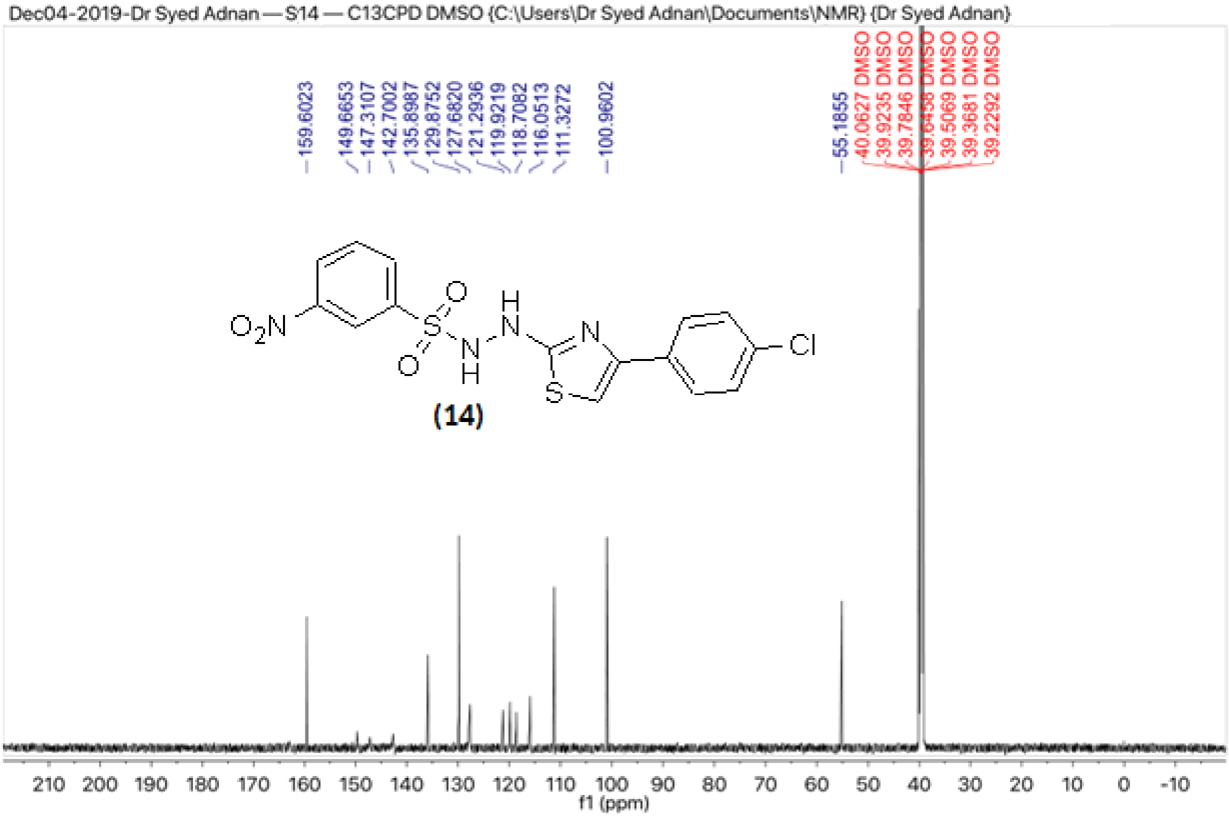

Figure-S18: Represent CNMR of analog 14

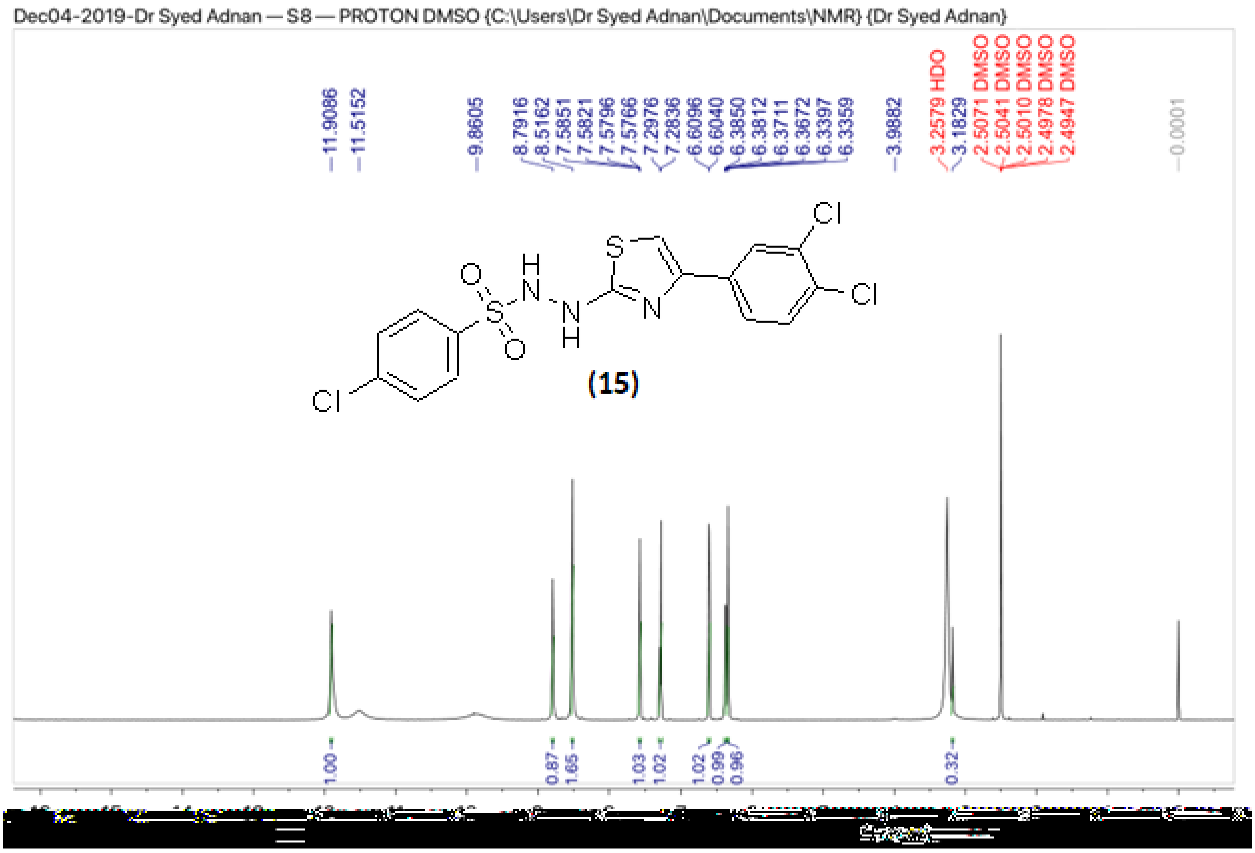

Figure-S19: Represent HNMR of analog 15

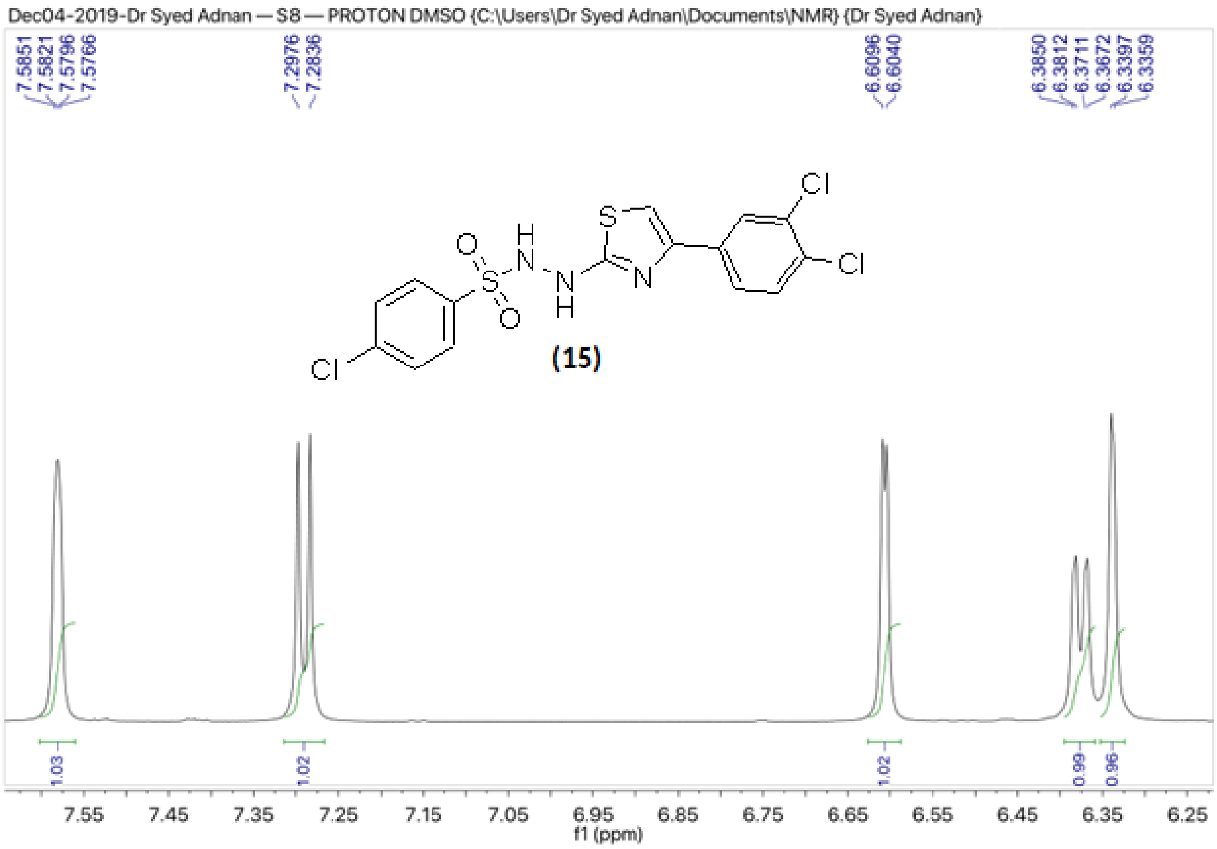

Figure-S20: Represent HR-HNMR of analog 15

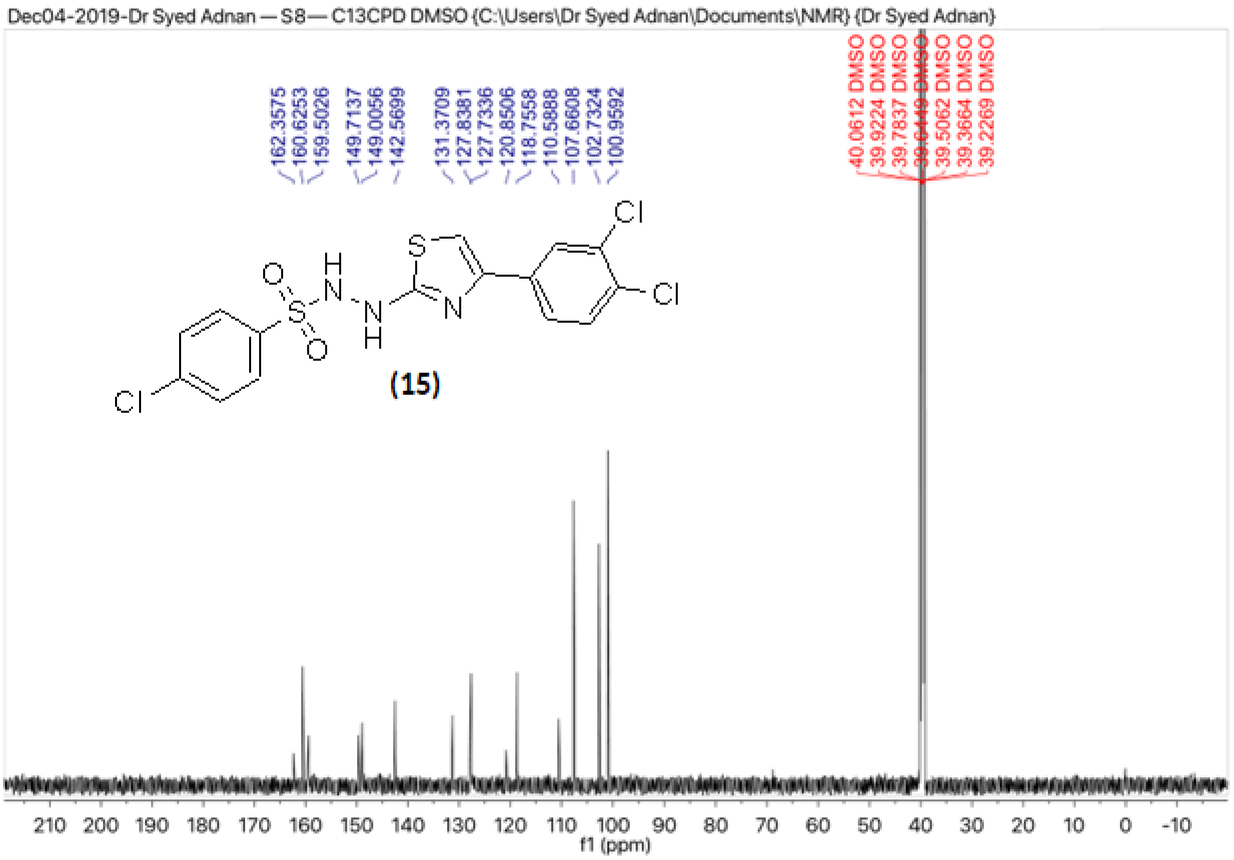

Figure-S21: Represent CNMR of analog 15

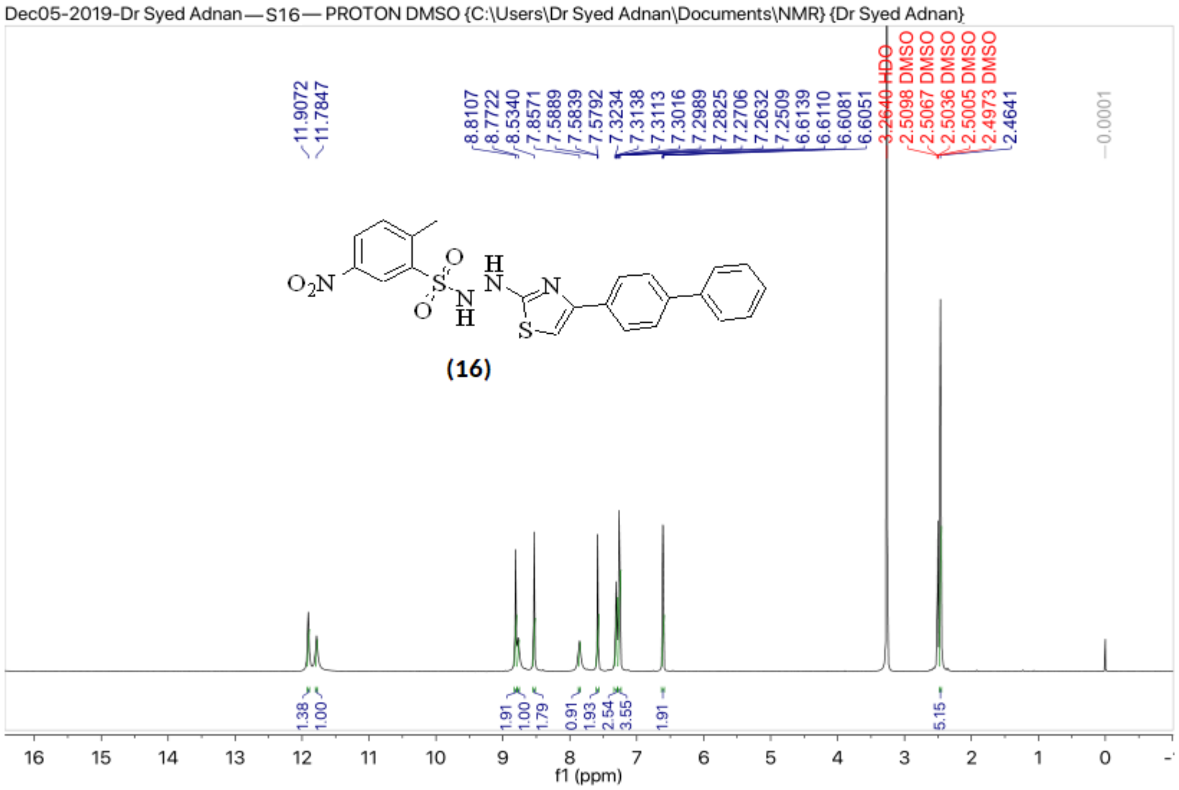

Figure-S22: Represent HNMR of analog 16

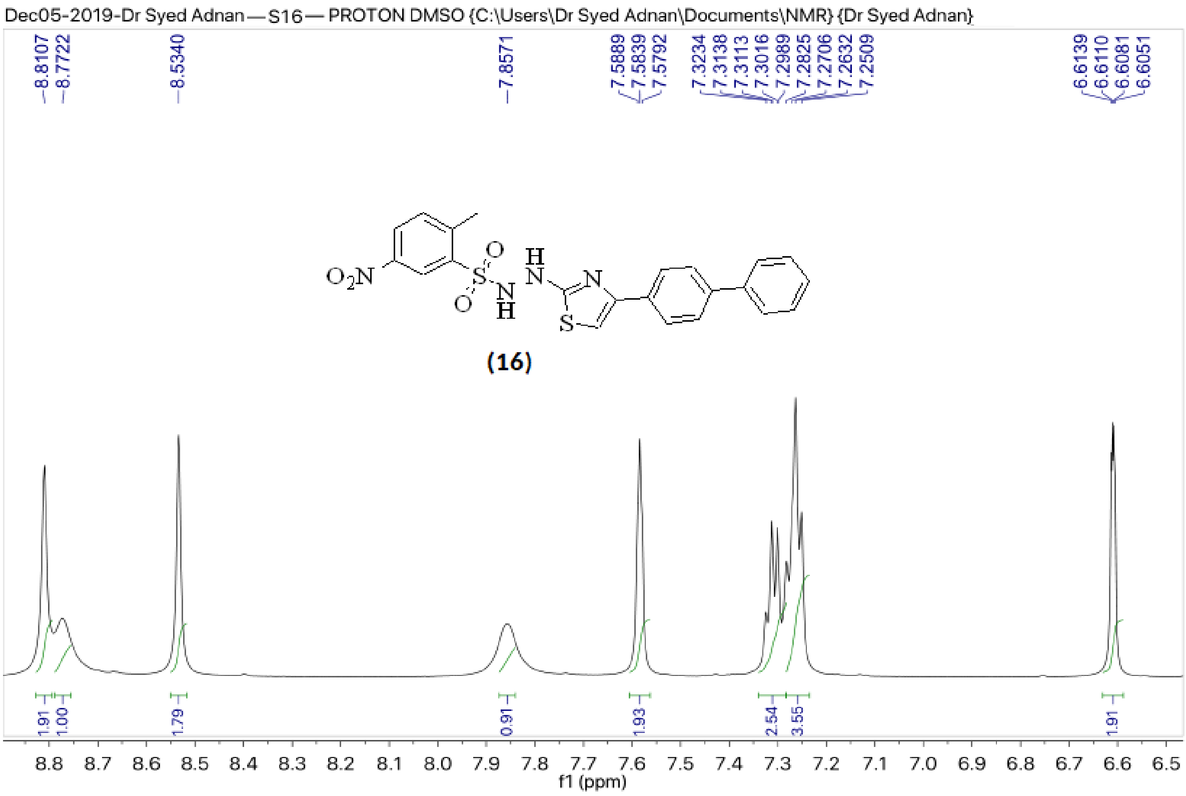

Figure-S23: Represent HR-HNMR of analog 16

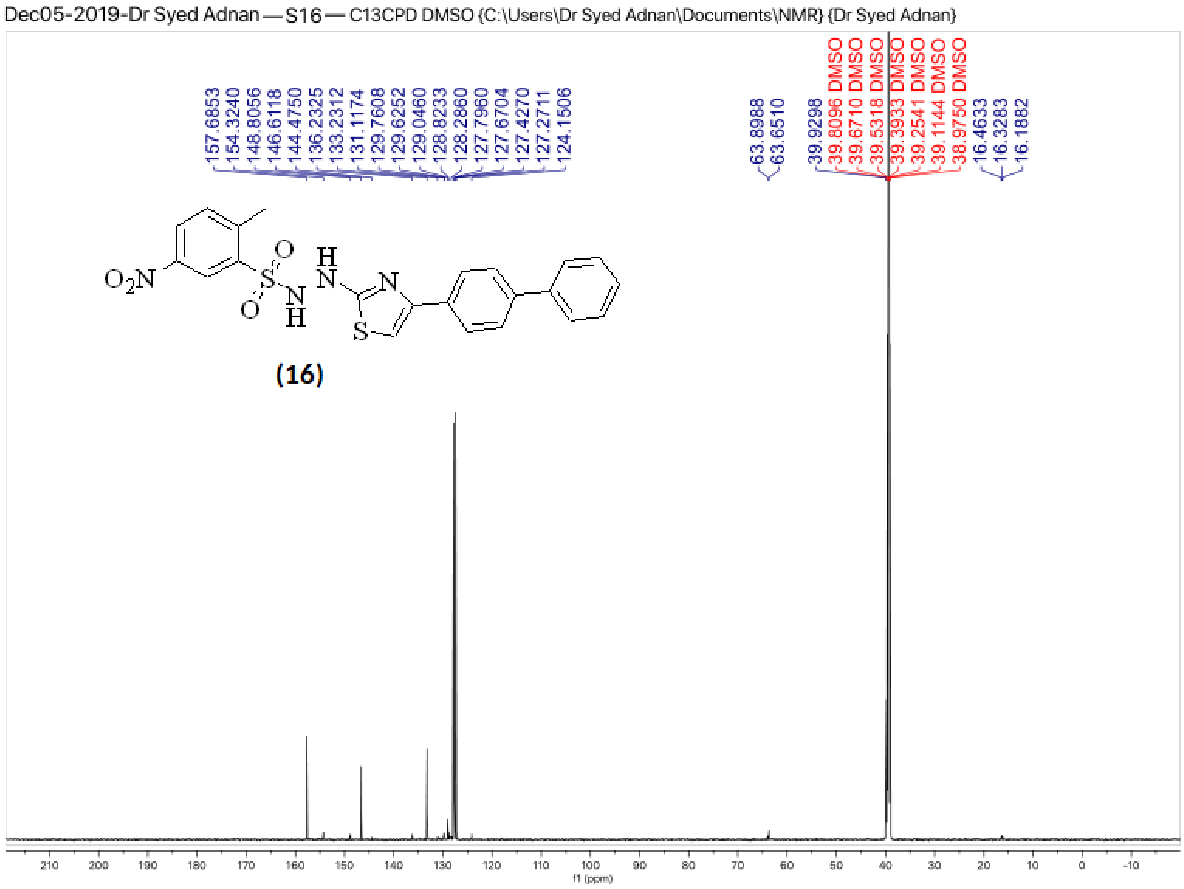

Figure-S24: Represent CNMR of analog 16
